# Blood alcohol levels in Finnish victims of non-ischaemic sudden cardiac death

**DOI:** 10.1080/07853890.2021.1890204

**Published:** 2021-03-01

**Authors:** Janna P. Kauppila, Lasse Pakanen, Katja Porvari, Juha Vähätalo, Lauri Holmström, Juha S. Perkiömäki, Heikki V. Huikuri, M. Juhani Junttila

**Affiliations:** a Research Unit of Internal Medicine, Medical Research Center Oulu, Oulu University Hospital and University of Oulu, Oulu, Finland; b Forensic Medicine Unit, Finnish Institute for Health and Welfare, Oulu, Finland; c Department of Forensic Medicine, Research Unit of Internal Medicine, Medical Research Center Oulu, University of Oulu, Oulu, Finland

**Keywords:** Alcohol, non-ischaemic heart disease, sudden cardiac death, medico-legal autopsy

## Abstract

**Introduction:**

Non-ischaemic heart disease (NIHD) is the underlying pathology in∼20% of all sudden cardiac deaths (SCDs). Heavy drinking is known to be associated with SCD due to ischaemic heart disease, but studies on association of recent alcohol consumption and SCD in patients with NIHD are scarce. We evaluated the blood alcohol levels of autopsy verified non-ischaemic SCD victims.

**Methods:**

Study population was derived from the Finnish Genetic Study of Arrhythmic Events (Fingesture) (*n* = 5869, mean age 65 ± 12, 79% males). All deaths occurred in Northern Finland during 1998–2017. All victims underwent a medico-legal autopsy. Subjects of SCD due to ischaemic heart disease were excluded.

**Results:**

A total of 1301 (mean age 57 ± 12, 78% males) victims of SCD due to NIHD were included in the study. The blood ethanol level was elevated in 543 (42%) subjects, out of which the blood alcohol level was ≥0.10%in 339 (62%) subjects and ≥0.15%in 252 (46%) subjects. Male SCD victims had alcohol in blood more frequently compared to females (45% versus 31%, *p* < .001).

**Conclusion:**

Elevated blood alcohol level is common in SCD victims due to NIHD, especially in males. Recent alcohol consumption might contribute to the subsequent SCD in many non-ischaemic SCD victims.

**KEY MESSAGES**
- Elevated blood alcohol level is common in victims of sudden cardiac death due to non-ischaemic heart disease, especially in males.
- Recent alcohol consumption may contribute to the subsequent death in many nonischemic sudden cardiac death victims.

## Introduction

Non-ischaemic heart disease (NIHD) consists of a diverse group of diseases, such as cardiomyopathies and inherited ion channel disorders, and accounts for about 20% of all sudden cardiac deaths (SCDs) [1]. The incidence of SCD in general and SCD from ischaemic heart disease seems to be declining, whereas the incidence of SCD from NIHD may have remained in the same level or even increased [2,3]. The three-year mortality rate of patients with severe NIHD with decreased left ventricular systolic function is approximately 12–20% [4,5]. Chronic heavy drinking can lead to alcoholic cardiomyopathy (CM), which is a common subtype of NIHD [1,6].

The role of alcohol in the development of SCD is a controversial subject. High alcohol consumption has long been known as a risk factor for SCD [7]. Light-to-moderate alcohol use has been reported to increase cardiovascular health and reduce the risk for SCD, [8,9]. but these findings have also been criticised [10]. Heavy drinking and especially binge drinking has been associated with SCD in patients with coronary artery disease in multiple studies [7,11]. Perkiömäki et al reported elevated blood alcohol levels in 38% of 1691 autopsied victims of SCD due to ischaemic heart disease [12]. While the risk for SCD due to heavy drinking is well established in patients with coronary artery disease, the association of recent alcohol consumption with SCD in patients with NIHD has not been largely studied. Our aim was to determine the blood alcohol levels of autopsy verified NIHD SCD victims.

## Materials and methods

**Figure 1.**
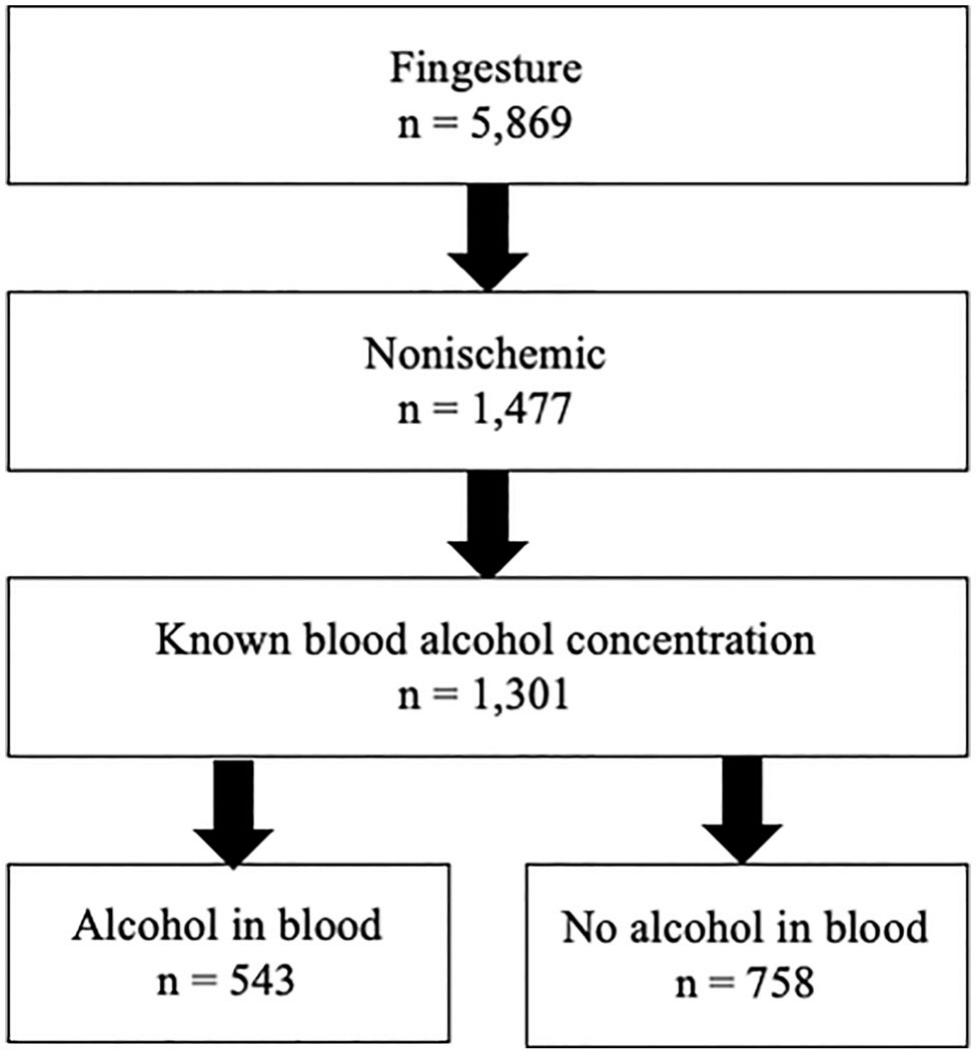
Flow chart of the study population.

Study population was derived retrospectively from the Finnish Genetic Study of Arrhythmic Events (Fingesture) which consists of 5869 (mean age 65 ± 12, 79% males) victims of SCD due to cardiac causes. This cohort study was designed to systematically collect information of all SCDs occurring in Province of Oulu, Northern Finland. All victims died between years 1998 and 2017 and were autopsied by experienced forensic pathologists. The medico-legal autopsies were performed at the Department of Forensic Medicine of the University of Oulu and Finnish Institute for Health and Welfare, Oulu, Finland. According to Finnish law, medico-legal autopsy is to be performed to all victims of sudden death if the patient has not been treated by physicians during their last illness, if the death is not due to a pre-existing known disease or it is otherwise unexpected. A death was considered sudden if it was witnessed within 6 h of the onset of symptoms or the person was last seen alive less than 24 h ago. The criteria for sudden death used in the Fingesture have been discussed in our previous study by Haukilahti et al. [13]. All autopsies included histologic examination. Toxicologic investigation was performed in cases with suspicion of toxic exposure, and all cases of intoxication and alcohol poisoning were excluded. The blood ethanol level of the subjects was determined by post-mortem gas chromatography in the Forensic Toxicology Unit, Finnish Institute for Health and Welfare, Helsinki, Finland. Blood alcohol concentrations ≥0.02% were considered positive/elevated. The post-mortem time intervals in Finland are on average about 5 days from death to autopsy and 2-3 weeks from death to toxicology investigation [14]. The probability of ethanol synthesis or other post-mortem factors influencing the toxicology results is considered small. In this study, SCD is defined as a sudden death of cardiac cause, as the study population did not include any cases of sudden death due to non-cardiac causes. Cases of SCD due to ischaemic heart disease (*n* = 4392) and cases with no information on blood alcohol level (*n* = 176) were excluded. The selection of the study population is illustrated in Figure 1.

Data on the subjects was gathered from autopsy reports, which included a death certificate and previous medical records, and questionnaires to closest family members. The expression “known heavy drinker” was used of a subject if they consumed large amounts of alcohol regularly. This information was documented in previous medical records over time by multiple physicians during a clinical assessment of the patient. In Finland, the definition of high-risk alcohol consumption is more than 16 drinks per week for females and 24 for males. While no information about specific consumption or duration of alcohol use of the subjects was available retrospectively, these definitions were probably used as a reference for heavy drinking.

The causes of death were reported according to ICD-10 code classes, and the more detailed classification of NIHD was based on the post-mortem findings being in conjunction with medical records and questionnaires. The underlying heart disease was known before death in only 33% of the cases. The subtypes of NIHD were classified as hypertensive CM, CM related to obesity, alcoholic CM, fibrotic CM, dilated CM, hypertrophic CM, myocarditis, valvular heart disease, arrhythmogenic right ventricular CM, anomalous coronary arteries, unspecified CM and structurally normal heart. Victims with a structurally normal heart were tested for long QT syndrome mutations. The definitions of these subtypes of NIHD are shown in Table 1 and have also been described in earlier Fingesture studies [1]. Stages of fatty liver and hepatic cirrhosis were determined during autopsy in a macroscopic and/or microscopic examination of the liver.

**Table 1.** Autopsy-based definitions of the subtypes of nonischemic heart disease (NIHD).

Causes of nonischemic SCD	Definition of the subtype of NIHD based on autopsy
Hypertensive CM	LVH, increased heart weight, unspecific fibrosis, other organ changes related to hypertension (e.g. arterial medial hypertrophy, intimal fibrosis in renal arterioles).
CM related to obesity	LVH or both left and right ventricular wall hypertrophy, increased heart weight, dilation of ventricles and atria, excessive epicardial and myocardial fat, obesity.
Alcoholic CM or unspecified CM	Focal replacement fibrosis of the myocardium, increased heart weight and LVH. In later stages, signs of dilated cardiomyopathy, other organ changes related to excessive long-term alcohol consumption (e.g. liver cirrhosis and/or severe steatosis, pancreatic fibrosis).
Fibrotic CM	Diffuse, interstitial or patchy myocardial fibrosis without myocardial scarring, LVH, or other apparent cause for fibrosis.
DCM	Left ventricular dilation with inadequate degree of LVH, unspecific fibrosis and focal atrophy or hypertrophy of myocytes. In later stages, pale and flabby myocardium and dilation of ventricles and atria.
HCM	Concentric LVH with myocyte disarray and various degrees of interstitial fibrosis, often asymmetrical septal hypertrophy.
Myocarditis	Inflammatory infiltration of the myocardium with necrosis and/or degeneration of the adjacent myocytes.
Valvular heart disease	Aortic or mitral valve calcification
ARVC	Right ventricular dilation, right ventricular myocardial atrophy with fibro fatty replacement of myocytes.
Anomalous coronary arteries	Anomalies of origination, course or coronary termination. Anomalous collateral vessels, anomalies of intrinsic coronary arterial anatomy.
Structurally normal heart	Macroscopically and microscopically normal heart with or without long QT syndrome mutations

ARVC: Arrhythmogenic right ventricular cardiomyopathy; CM: cardiomyopathy; DCM: dilated cardiomyopathy; HCM: hypertrophic cardiomyopathy, LVH: left ventricular hypertrophy.

The study complies with the Declaration of Helsinki and was approved by the ethics committee of Northern Ostrobothnia Hospital district (Oulu University Hospital). Permissions to review the medico-legal autopsy data were gained from the Finnish National Institute for Health and Welfare and the Regional State Administrative Agency of Northern Finland.

### Statistical analysis

Chi-square test was used to detect significant differences in the distribution of dichotomised variables and Fisher’s exact test was used when a cell count was less than five, and thus, the assumptions for validity of the Chi-square test were violated. Gaussian distribution of variables was evaluated by skewness test. When comparing continuous variables of two groups, independent samples t-test was used for variables with normal distribution and Mann–Whitney test for variables with non-normal distribution. The IBM Statistical Package for Social Studies 25 (SPSS Inc., Chicago, IL) was used to perform the analyses, and two-sided *p*-values <.05 were considered statistically significant.

## Results

**Figure 2.**
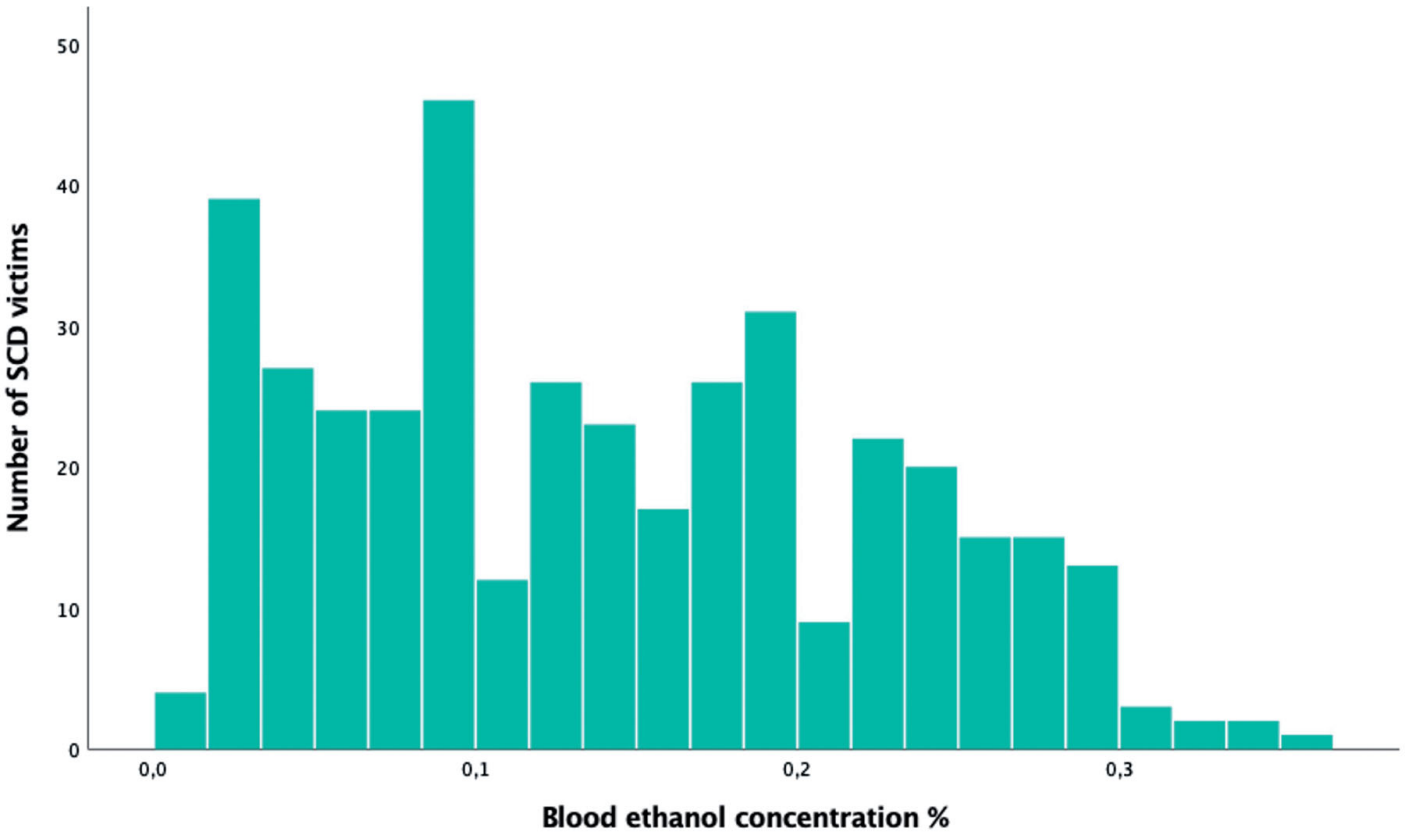
Blood ethanol concentration in 543 victims of sudden cardiac death(SCD) with elevated blood alcohol level.

A total of 1301 (mean age 57 ± 12, 78% males) victims of SCD due to NIHD were included in the study. .The characteristics of the study population are shown in Table 2. The blood ethanol level was elevated in 543 (42%) subjects, out of which the concentration of alcohol was ≥0.10% in 339 (62%) subjects and ≥0.15% in 252 (46%) subjects. The alcohol concentration of subjects with elevated blood ethanol level is shown in Figure 2. The group with alcohol in blood had more males than the group with no alcohol in blood, and male SCD victims had more frequently alcohol in blood compared to females (45% versus 31%, *p* < .001). Among males with alcohol in blood, the concentration of alcohol was ≥0.10%in 282 (62%) subjects and ≥0.15% in 213 (47%) subjects. Among females with alcohol in blood, the concentration of alcohol was ≥0.10% in 57 (63%) subjects and ≥0.15% in 39 (43%) subjects. The medians of concentrations of alcohol between males with alcohol in blood and females with alcohol in blood were similar (0.14% versus 0.13%, *p* = .476). Out of all victims of NIHD SCD, 664 (51%) were known to be heavy drinkers. Subjects with alcohol in blood were more often known heavy drinkers than subjects with no alcohol in blood (61% versus 44%, *p* < .001). Within known heavy drinkers (*n* = 664), alcohol blood test was positive in 329 (50%).

**Table 2.** Characteristics of the study population and subgroups.

	Total *n* = 1,301	Victims with alcohol in blood *n* = 543 (42 %)	Victims with no alcohol in blood *n* = 758 (58 %)	*p* Value
Age, years (mean ± standard de*via*tion)	57 ± 12	58 ± 11	56 ± 13	.067
Gender, male	78 %	83 %	74 %	<.001
BMI, kg/m**^2^** (mean ± standard de*via*tion)	30 ± 8.1	30 ± 7.8	30 ± 8.3	.747
Diabetes, %	19 %	20 %	19 %	.925
Hypertension^a^, %	42 %	47 %	39 %	.007
Moderate or severe fatty liver in autopsy^b^	57 %	63 %	53 %	<.001
Any stage of hepatic cirrhosis in autopsy^b^	34 %	37 %	32 %	.067
Time of death (24-06/06-12/12-18/18-24/unknown) %	9/8/7/7/69	9/7/5/7/72	9/9/8/7/68	.293
Hypertensive CM *n* = 351	8/6/7/8/71	9/5/5/9/72	7/7/8/7/70	.489
CM related to obesity *n* = 307	13/9/12/9/57	10/10/10/12/58	16/8/13/7/56	.161
Alcoholic CM *n* = 289	5/6/4/2/83	4/5/4/2/84	6/6/4/3/82	.994
Fibrotic CM *n* = 170	11/6/6/5/73	14/3/2/0/81	9/7/8/7/69	.050

BMI: body mass index; CM: cardiomyopathy.

^a^Missing data from 39 subjects.

^b^Missing data from 1 subject.

The causes of SCD due to NIHD are presented in Table 3. The most prevalent causes of death were hypertensive CM (27%), CM related to obesity (24%), alcoholic CM (22%) and fibrotic CM (13%). Out of 13 subjects with structurally normal heart, long QT syndrome mutation was found in 4 (31%) subjects. When compared to other types of NIHD, hypertensive CM, fibrotic CM, myocarditis and valvular heart disease were associated with elevated blood alcohol level (Table 3).

**Table 3.** Types of underlying nonischemic heart disease (NIHD) in subjects with alcohol in blood and subjects with no alcohol in blood.

Causes of nonischemic SCD	All victims of nonischemic SCD *n* = 1,301 (%)	Victims with alcohol in blood *n* = 543 (42 %)	Victims with no alcohol in blood *n* = 758	*p* Value
Hypertensive CM	351 (27^a^)	162 (46^b^)	189 (54^c^)	.050
CM related to obesity	307 (24)	135 (44)	172 (56)	.363
Alcoholic CM	289 (22)	135 (47)	154 (53)	.052
Fibrotic CM	170 (13)	58 (34)	112 (66)	.031
DCM	39 (3)	13 (33)	26 (67)	.280
HCM	30 (2)	14 (47)	16 (53)	.580
Myocarditis	46 (4)	9 (20)	37 (80)	.002
Valvular heart disease	44 (3)	10 (23)	34 (77)	.009
ARVC	5 (0)	3 (60)	2 (40)	.655
Anomalous coronary arteries	4 (0)	0 (0)	4 (100)	.145
Unspecified CM	3 (0)	0 (0)	3 (100)	.270
Structurally normal heart	13 (1)	4 (31)	9 (69)	.575

ARVC: Arrhythmogenic right ventricular cardiomyopathy; CM: cardiomyopathy; DCM: dilated cardiomyopathy; HCM: hypertrophic cardiomyopathy.

^a^Percentage of cause of death within all victims.

^b^Percentage of cases with alcohol in blood.

^c^Percentage of cases with no alcohol in blood.

## Discussion

Elevated blood alcohol level was observed in more than 4 out of 10 victims of non-ischaemic SCD and was more common in males compared to females. Hypertensive CM, fibrotic CM, myocarditis and valvular heart disease were associated with a positive blood alcohol test.

Extensive research has been done on the effects of alcohol on the cardiovascular system. Acute alcohol intake increases blood pressure, promotes neurohormonal stimulation and causes left ventricular dysfunction, which may last for a few days even after withdrawing from alcohol [15,16]. As a part of the Fingesture study, Perkiömäki et al. [12] reported elevated blood alcohol levels in 38% of 1,691 autopsied victims of SCD due to ischaemic heart disease, suggesting an association between acute alcohol intake and increased risk of SCD in patients with coronary artery disease. Sjögren et al.^17^ demonstrated elevated blood alcohol levels in 39% of 15,630 autopsied victims of all types of unnatural deaths. Chronic drinking seems to have both favourable and harmful effects on the heart. Light-to-moderate alcohol consumption (two to six standard drinks per week) has been reported to lower blood pressure and have beneficial effects on haemostatic factors, lipoproteins and inflammatory markers, lowering the risk of coronary artery disease, myocardial infarction, and death from cardiovascular disease [16,18]. However, some studies have been criticized for classifying ex-drinkers in the same category as non-drinkers, which might result in a bias towards light-to-moderate drinkers [10]. Chronic heavy drinking (more than six standard drinks per day) is associated with hypertension, myocyte hypertrophy and increased cortisol and cholesterol levels, increasing the risk of myocardial infarction, dilated CM, heart failure and cardiac arrhythmias [7,16,19]. Binge drinking, or heavy episodic drinking, is associated with myocardial inflammation and arrhythmias, such as atrial fibrillation, ventricular tachycardia and ventricular fibrillation [6].

NIHD consists of various different conditions, such as CMs, myocarditis and valvular heart disease. CMs compose the majority of NIHD [1]. Inherited ion channel disorders, such as long QT syndrome, are a type of NIHD and one possible explanation for SCD in victims with a structurally normal heart in autopsy [1]. The three-year mortality rate of severe NIHD with reduced left ventricular ejection fraction (<35%) is approximately 12–20%, with death resulting typically from ventricular arrhythmias or heart failure [4,5]. NIHD is responsible for about 30–40% cases of heart failure [20]. Patients with CMs develop progressive heart failure and structural changes, such as hypertrophy, fatty or fibrotic infiltration, chamber dilation and reduced ejection fraction, as well as tachyarrhythmias, which may lead to SCD [21]. In this study, the most prevalent causes of non-ischaemic SCD were hypertensive CM, CM related to obesity, alcoholic CM and fibrotic CM.

An estimated 30–50% of all non-ischaemic CMs is associated with alcohol abuse [16]. After genetic factors, alcohol abuse is a leading cause of non-ischaemic dilated CM [6,16]. Chronic alcohol abuse reduces cardiac contractility, activates compensatory mechanisms and can thus lead to alcoholic CM, which is characterised by ventricle dilation, left ventricular hypertrophy and reduced ejection fraction [6,16,19]. It is estimated that a consumption of about 7–8 standard drinks per day for >5 years is a significant risk for developing alcoholic CM in both males and females [19]. Nevertheless, less than half of all chronic heavy drinkers develop alcoholic CM [1,16,19]. The prevalence of alcoholic CM is higher in men compared to women, as is death from alcoholic CM [16,19]. Men drink more alcohol than women, however, women might be more vulnerable to the effects of alcohol abuse and to developing alcoholic CM [6,19]. In our study, elevated blood ethanol level at the time of SCD was more often seen in men than women.

The majority of studies concerning alcohol ingestion and SCD have been done on ischaemic heart disease. The effect of alcohol on the development of alcoholic CM is well established, but information on the association of alcohol abuse with non-ischaemic SCD is limited. In a large, population-based study by Klatsky et al. [9], heavy alcohol abuse was reported to increase the risk of heart failure in patients with no coronary artery disease. Cooper et al. [8] found no association between light-to-moderate drinking and death from NIHD in patients with left ventricular systolic dysfunction, although a modest trend towards risk of hospitalisation from non-ischaemic heart failure was seen. In an earlier study of 7,735 middle-aged British men, Wannamethee et al. [7] demonstrated a marginally increased risk of sudden death in heavy drinking men with no evidence of pre-existing coronary artery disease. In their study, cause of death was defined only by existing information on medical history, as the victims were not autopsied; also, the study included all types of sudden death. Cittadini et al. [22] released a case report about an autopsy-verified victim of SCD due to arrhythmogenic right ventricular CM and ethanol and cocaine abuse. Alcohol abuse can prolong the QT interval and has been reported to increase the risk of drowning in patients with long QT syndrome [23].

Our study suggests a possible association between acute alcohol intake and autopsy-verified non-ischaemic SCD. As previous research data concerning non-ischaemic SCD and alcohol is limited, it is not quite obvious why hypertensive CM, fibrotic CM, myocarditis and valvular heart disease specifically were associated with elevated blood alcohol level compared to other types of NIHD. We found no studies about alcohol use in patients with myocarditis or valvular heart disease as a cause of death. Hypertensive and fibrotic CM, on the other hand, often cause changes in the myocardium. Therefore, a possible mechanism behind the associations mentioned before could be a synergic effect of the arrhythmia-inducing structural abnormalities and reduced cardiac contractility often seen in NIHD, with the susceptibility to arrhythmias and left ventricular dysfunction caused by acute alcohol intake. Further studies are needed to assess whether this association could be described as a causal effect, as well as to determine the relationship between chronic drinking and the risk of non-ischaemic SCD.

Limited data are available about alcohol consumption as a contributor to death in Finland. According to Statistics Finland (2018), alcohol-related disease and alcohol poisoning account for about 3% of all deaths in Finland, although alcohol-related deaths may be slightly under-recorded [24]. A medico-legal autopsy is performed in 15–20% of deaths that occur in Finland (Statistics Finland 2015), and toxicologic investigation is performed in most cases. Ketola et al. reported elevated blood alcohol levels in 36% of 122 234 Finnish autopsy cases over 18 years [25]. The majority (78%) of the subjects were men, and male victims had significantly more often alcohol in blood compared to females (81% vs 19%). These findings do not represent the whole population and are biased towards users of alcohol, as toxicologic investigation is performed primarily in cases with suspicion of toxic exposure. Also, in our study, all cases of intoxication and alcohol poisoning were excluded.

In previous studies, the cause of a sudden death has often been determined by prior medical history. In this study, all deaths were confirmed to be due to a cardiac cause by autopsy. We consider autopsy to be a more reliable way to determine the cause of a sudden death, for many sudden conditions, such as aortic dissection, pulmonary embolism and stroke, can lead to sudden death and might be interpreted as SCD, if not autopsied [26].

## Limitations

As a limitation, this study only addresses acute alcohol intake at the time of SCD, as previous use of alcohol was not taken into account. Almost half of the subjects with no alcohol in blood were known to be heavy drinkers, although the subjects with alcohol in blood were more often known heavy drinkers. In addition, toxicologic investigation was not performed to all victims of SCD, presenting a possible bias towards cases in which alcohol consumption might have been suspected. Also, medico-legal autopsy is performed with a higher probability if the victim is a known heavy drinker, which might bias the results towards the group with alcohol in blood. No control population could be included in the study, and it is debateable whether these findings can be generalised to other populations. Nevertheless, we consider the autopsy verification of all cases of SCD a major strength in the study, offering an accurate way to determine the causes of non-ischaemic SCD.

## Conclusions

Elevated blood alcohol level is common in victims of SCD due to NIHD, especially in males. Recent alcohol consumption may contribute to the subsequent SCD in many non-ischaemic SCD victims.

## Data Availability

The data is available for reproduction of results on request from the corresponding author.
